# Characterization of a Pathogenic Variant in the ABCD1 Gene Through Protein Molecular Modeling

**DOI:** 10.1155/2020/3256539

**Published:** 2020-01-25

**Authors:** John E. Richter Jr., Charitha Vadlamudi, Sarah K. Macklin, Ayesha Samreen, Haytham Helmi, Daniel Broderick, Ahmed N. Mohammad, Stephanie L. Hines, Jay A. VanGerpen, Paldeep S. Atwal, Thomas R. Caulfield

**Affiliations:** ^1^Department of Clinical Genomics, Mayo Clinic, Jacksonville, FL 32224, USA; ^2^Department of Pulmonology, Mayo Clinic, Jacksonville, FL 32224, USA; ^3^Department of Endocrinology, Mayo Clinic, Jacksonville, FL 32224, USA; ^4^Department of Radiology, Mayo Clinic, Jacksonville, FL 32224, USA; ^5^Department of Internal Medicine, Mayo Clinic, Jacksonville, FL 32224, USA; ^6^Department of Neurology, Mayo Clinic, Jacksonville, FL 32224, USA; ^7^Department of Neuroscience, Mayo Clinic, Jacksonville, FL 32224, USA; ^8^Department of Cancer Biology, Mayo Clinic, Jacksonville, FL 32224, USA; ^9^Department of Neurosurgery, Mayo Clinic, Jacksonville, FL 32224, USA; ^10^Department of Health Sciences Research, Mayo Clinic, Jacksonville, FL 32224, USA

## Abstract

**Background:**

The ATP-binding cassette, subfamily D, member 1 (ABCD1) protein is a peroxisomal half-transporter that allows for very long chain fatty acid (VLCFA) degradation. Pathogenic variants of *ABCD1* cause VLCFAs to build up in various tissues and bodily fluids, resulting in a disorder called X-linked adrenoleukodystrophy (X-ALD). This disorder is most commonly marked by adrenocortical insufficiency and high VLCFA concentration, and has varying levels of neurological involvement depending on phenotype. For example, the Addison-only form of X-ALD has no neurological impact, while the cerebral form of X-ALD often causes severe sensory loss, motor function impairment, cognitive decline, and death.

**Methods:**

A newly characterized and suspected pathogenic variant in *ABCD1* cause VLCFAs to build up in various tissues and bodily fluids, resulting in a disorder called X-linked adrenoleukodystrophy (X-ALD). This disorder is most commonly marked by adrenocortical insufficiency and high VLCFA concentration, and has varying levels of neurological involvement depending on phenotype. For example, the Addison-only form of X-ALD has no neurological impact, while the cerebral form of X-ALD often causes severe sensory loss, motor function impairment, cognitive decline, and death.

**Results:**

A case of adult onset adrenomyeloneuropathy (AMN) and a novel *ABCD1* cause VLCFAs to build up in various tissues and bodily fluids, resulting in a disorder called X-linked adrenoleukodystrophy (X-ALD). This disorder is most commonly marked by adrenocortical insufficiency and high VLCFA concentration, and has varying levels of neurological involvement depending on phenotype. For example, the Addison-only form of X-ALD has no neurological impact, while the cerebral form of X-ALD often causes severe sensory loss, motor function impairment, cognitive decline, and death.

**Conclusions:**

Data fusion from multiple sources was combined in a comprehensive approach yielding an enriched assessment of the patient's disease and prognosis. Molecular modeling was performed on the variant to better characterize its clinical significance and confirm pathogenicity.

## 1. Introduction

The ATP-binding cassette, subfamily D, member 1 (ABCD1) protein is part of the ATP-binding cassette transporter superfamily, which consists of transmembrane proteins responsible for passing lipids, metabolites, and other molecules between cells or intracellular structures. It is encoded by *ABCD1*, a gene located at Xq28 [OMIM#300371]. ABCD1 is a peroxisomal half-transporter that imports very long chain fatty acids (VLCFA) into the peroxisome by binding ATP. Dysfunctional ABCD1 is unable to move VLCFAs to the peroxisome to be degraded, resulting in VLCFA accumulation in body fluids and tissues [1]. This abnormal level of VLCFAs serves as a characteristic biomarker of X-linked adrenoleukodystrophy (X-ALD), the most common peroxisomal disorder [2]. X-ALD is a genetic condition primarily associated with demyelination, neurodegeneration, and adrenocortical insufficiency [3]. Over 800 variants have been reported within the *ABCD1* gene [ALD Mutation Database]. Despite this, clear genotype–phenotype correlations have not been observed [4].

Hemizygous males typically exhibit the most significant symptoms, while heterozygous females often develop a lesser degree of disability later in life [2]. The three classic phenotypes are Addison disease-only, adrenomyeloneuropathy (AMN), and cerebral ALD. Individuals with the Addison disease-only form usually present with adrenocortical insufficiency between 2 and 7 years old. Neurologic degeneration is not present initially, but it often develops as the individual ages [5]. AMN typically onsets in the third to fourth decade of life and primarily affects the spinal cord [3]. The presenting clinical feature is often progressive leg stiffness and weakness. Adrenal insufficiency is found in roughly 2/3 of AMN patients, and cerebral changes affect around half of them [3]. Cerebral ALD is the most severe phenotype, where cerebral demyelination causes rapid decline in hearing, sight, motor function, and cognition [5]. Most commonly found in children or adolescents, this variant of X-ALD often results in death within 2–4 years of symptom presentation [2].

This report details the history of a proband diagnosed in his thirties with AMN. Molecular analysis of the *ABCD1* gene identified a variant of uncertain significance within this individual. Interestingly, this variant has been reported pathogenic in the ALD Mutation Database, but information regarding the clinical case was not published. Our proband's clinical case is included to better support the pathogenicity of this *ABCD1* variant. Protein informatics platform (PIP) was utilized for comprehensive molecular modeling to further characterize the significance of this variant and clarify to the proband's diagnosis.

## 2. Case Study (Clinical Description)

The proband was a 31-year-old male who presented for evaluation of a progressive neurologic condition. Symptoms of this condition first appeared 2 years ago when the proband began to trip increasingly often. He would later experience generalized muscle weakness and stiffness, along with further gait dysfunction. To combat this, the proband had used a rolling walker to aid in ambulation for the past 7 months. He had also developed urinary incontinence. Furthermore, he reported brain fog, memory loss, attention deficits, anxiety, and fatigue. Basic mental tasks took longer to complete as a result. Per the proband, alcohol use exacerbated these deficits. Finally, the proband lost 20 pounds since the onset of his condition and was unable to regain weight despite concerted efforts to do so. This weight loss was partially due to symptoms of nausea and vomiting, which were most common in the morning.

During physical examination, the proband was unable to rise from a sitting position without the use of his hands. Mild ataxic dysarthria was identified. Visual fixation tests revealed infrequent square wave jerks. Smooth pursuits were saccadic, and he demonstrated slow, full saccades both vertically and horizontally. Stretch reflexes were pathologically brisk in the upper extremities. The proband also had pathologically brisk mitotic stretch reflexes in the lower extremities, including sustained clonus at the ankles. Moderately impaired proprioception of the great toes was present. Mild upper motor neuron-distribution weakness was seen in the lower extremities with sparing of the iliopsoas. Heel-to-shin testing revealed dyssynergia. While ambulating, the proband looked at his feet and moved with a reduced stride. His knees hyperextended, he tended to toe walk, and occasionally he slid his feet. Romberg sign was present. While standing, tremulous movements were present in his legs—predominantly in the quadriceps. The proband turned 360 degrees over 3 trials, which required 9 to 15 steps per turn. His postural reflexes were abnormal, and the “Pull” test was −3.

**Figure 1:**
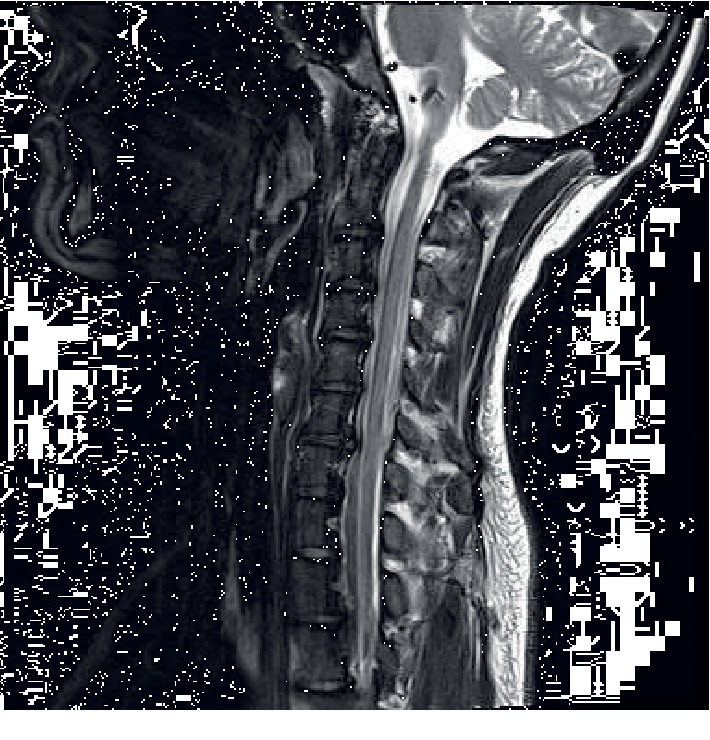
MRI of cervical spine. MRI of the cervical spine revealed findings suggestive of mild diffuse spinal cord atrophy.

**Figure 2:**
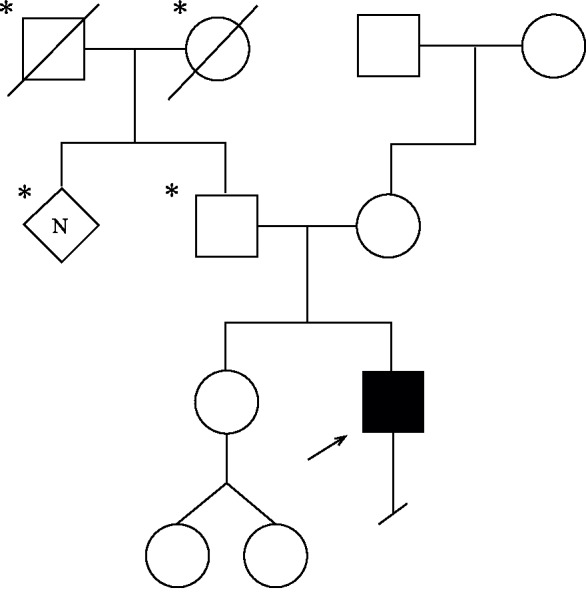
Family pedigree. Shaded individuals had reported history of gait dysfunction. ^∗^Indicates limited medical history known. The proband is marked with an arrow.

MRI of the brain was normal, and MRI of the cervical spine revealed findings suggestive of mild, diffuse spinal cord atrophy. There was no evidence for subacute combined degeneration or other acquired spinal cord disease (Figure 1). He had been treated for Lyme disease, as his Lyme IgM titers were positive, but no significant improvement had been recorded. His cerebrospinal fluid was described as acellular. Evaluation of HIV, HTLV1, hepatitis C, hepatitis B, and cytomegalovirus returned negative. Family history of similar symptoms could not be established, as the proband's knowledge of their medical histories was limited (Figure 2).

The proband's peroxisomal fatty acid profile was obtained after the previous tests and imaging. Abnormal concentrations of C26 : 0, C24/C22, and C26/C22 were present, which suggested hemizygosity for X-linked adrenomyeloneuropathy. Adrenocorticotropic hormone measured 528 pg/mL (RR: 7.2–63). Sequencing and deletion/duplication analysis of the *ABCD1* gene was then completed. The proband was hemizygous for a variant of uncertain significance in *ABCD1*, c.1599G>T (p.Lys533Asn). This variant was not present in the ExAC online database, though it was listed as pathogenic in the ALD Mutation Database. Two similar variants, c.1596A>G (p.Lys533Glu) and c.1598A>G (p.Lys533Arg), have also been reported previously as clinically significant [6, 7].

## 3. Methods

### 3.1. Structural Modeling

The sequence of human ATP-binding cassette subfamily D member 1 (known as ABCD1) is likely a transporter protein, which has a nucleotide-binding region with a fold that can act as an ATP-binding subunit with ATPase activity. It is known that correct dimerization is required to form an active transporter. ABCD1 is a member of the ALD subfamily that takes part in peroxisomal fatty acid import into organelles. These peroxisomal ABC transporters are “half transporters,” meaning that they require a partner half transporter molecule—the functional form is always homodimeric or heterodimeric. ABCD1 is assumed to be the key for peroxisomal transport or catabolism of very long chain fatty acids. ABCD1 is known to interact with PEX19, and is encoded by the *ABCD1* gene, which was taken from the NCBI Reference Accession Sequence: NP_000024: version NP_000024.2, which is encoded for the amino acid sequence; and was used for computer assisted modeling. Monte Carlo simulations were performed on the mutant to allow local regional changes for full-length 745 amino acids.

The X-ray refinement for Monte Carlo was built using the YASARA SSP/PSSM Method [8–13]. The structure was relaxed to the YASARA/Amber force field using knowledge-based potentials within YASARA. The side chains and rotamers were adjusted with knowledge-based potentials, simulated annealing with explicit solvent, and small equilibration simulations using YASARA's refinement protocol [14]. The entire full-length structure was modeled, filling in any gaps or unresolved portions from the X-ray.

Refinement of the finalized models was completed using either the Schrodinger's LC-MOD Monte Carlo-based module or the NAMD2 protocols. These refinements started with YASARA generated initial refinement and variant [8–10, 12]. The superposition and subsequent refinement of the overlapping regions yields a complete model for the ABCD1. The final structures were subjected to energy optimization with PR conjugate gradient with an R-dependent dielectric.

Atom consistency was checked for all 745 amino acids (12,201 atoms) of the full-length wild-type model (WT) and 745 amino acids (12,221 atoms) for the variant, verifying correctness of chain name, dihedrals, angles, torsions, nonbonds, electrostatics, atom-typing, and parameters. A dimer model is predicted, which consists of 24,402 atoms including cofactors and ions. Each model was exported to the following formats: Maestro (MAE), and YASARA (PDB). Model manipulation was done with Maestro (Macromodel, version 9.8, Schrodinger, LLC, New York, NY, 2010), or Visual Molecular Dynamics (VMD) [15]. Analyses were emphasized on the N-terminus region containing the first 350 amino acids given the length and C-term distance from site of mutation.

Monte Carlo dynamics searching (hybrid MC or via enhance MDS sampling) was completed on each model for conformational sampling, using methods previously described in the literature [16–19]. Briefly, each ABCD1 variant system was minimized with relaxed restraints using either Steepest Descent or Conjugate Gradient PR, then allowed to undergo the MC search criteria, as shown in the literature [16–19]. The primary purpose of MC, in this scenario, is examining any conformational variability that may occur with different mutations in the region near to the mutation and the possible effect on DNA binding or processing with the ABCD1.

## 4. Results

### 4.1. Structure-Function Studies

**Figure 3:**
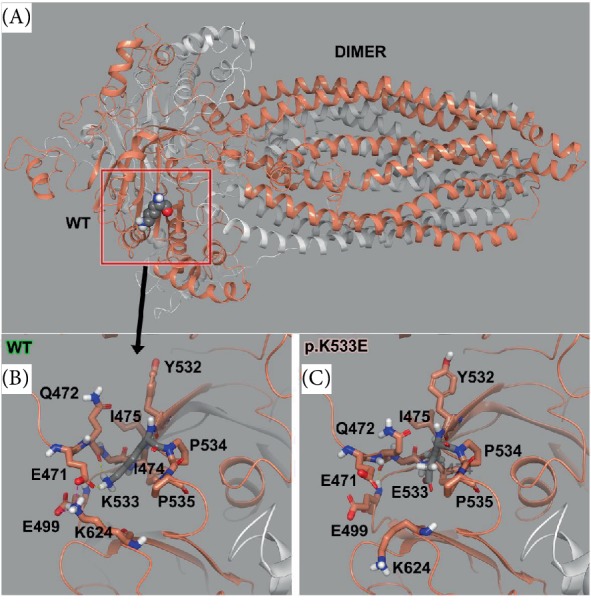
ABCD1 molecular model for full-length human sequence for variant p.K533E. (A) Full-length dimeric model for the entire wild type (WT) ABCD1 structure shown as ribbon structure with VdW sticks for position 533. (B) Zoom-in on the wild-type for the Lys533 residue from the full-length model to better show the interacting residues and dimer interface. Monomer 1 is gray and monomer 2 is orange. The residues within interaction distance to K533 are shown and yellow dash line is H-bonds and pink dash line is salt bridges. (C) Zoom-in on the region around Glu533 variant, showing nearby residues and the resulting distortion of the “pocket” around E533 due to the charge flipping (+ to −).

For WT versus the variant p.K533E, we found the stability of the object from energetic calculations for ΔG per amino acid to remain relatively the same, such that WT has an object stability of 114.67 kcal/mol^∗^Å^2^. The variant p.K533E causes a net increase in free energy of 2.321 kcal/aa^∗^mol^∗^Å^2^, which could be destabilizing to the local region [16–18, 20–23]. This object stability was positive indicating that some dynamic changes are likely with a molecular simulation for conformational sampling. Thus we examined the local residues and determined an electrostatic calculation may be useful to explain the change in function. The molecular model for the full structure and its variant form are given (Figure 3(a)) using our state of the art methods, which have been established [16–20, 23–30]. The dimeric model is critical for function and shows important interactions at the site of mutation that are disrupted by the variant mutation.

**Figure 4:**
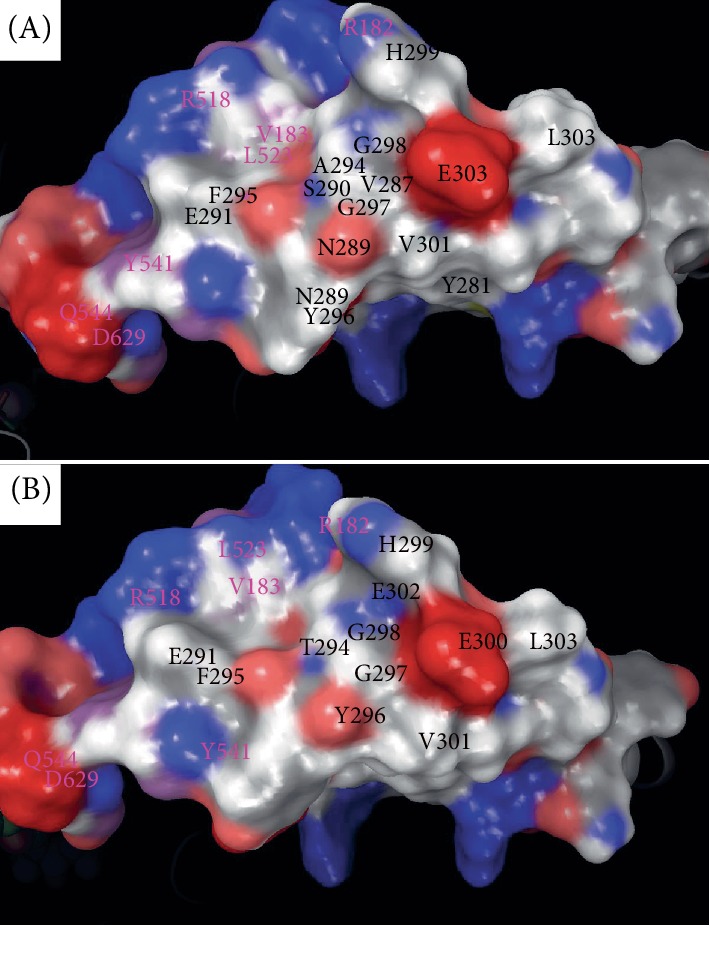
ABCD1 electrostatic mapping for interaction potential for the full-length model for the entire ABCD1 structure with electrostatics calculated using Poisson–Boltzman (PB) calculation overlaid onto structure. The relevant region is zoomed into showing all residues within 12 Å of residue position. (A) Interacting residues surrounding the residues are given with PB electrostatics mapped onto them. The black labels are for monomer 1 positions of amino acids nearby residues and purple label are given for monomer 2 positions of amino acids nearby to central residues. The electrostatic map shows the negative to positive (red to blue) distribution and the contours and shape of the protein in this region. (B) Mutant variant from ABCD1 model is given with electrostatics overlaid indicating changes in charge. The depiction is similar to (A).

Local residues within the 12 Å cutoff near the variant site (p.K533E) include residues from both monomers. The monomer containing the variant (monomer 1, gray carbons) has the following nearby residues with position 533-interactions: E471, Q472, I474, I475, E499, Y532, P534, P535, and K624 (Figure 3(b)). This variant structure (p.Lys533Glu) was analyzed and more straightforward disruption of the local environment was indicated from the positive lysine (+) charge flipping to a negative glutamic acid (−) that was quite disruptive to the adjacent charged residues (above). In particular, residues K624, E499, and Y532 are placed into different interactions (Figure 3(b)). Local ΔG per amino acid increases a net positive value corresponding to unfavorable stability in the pocket and longer dynamics would be required to determine the overall effect but can expected to be destabilizing. The K533E residue has interesting interactions with Q472, I474, I475, and E499. K533E is in a helix-helix region where its charged side chain is positioned outward to oppositely charged species; there is also some helix-helix interaction with the adjacent helices (Figures 3(b) and 3(c)). The same residues are implicated in the variant, but the position is changed for several of the residues including P534, P535, and K624 (Figure 3(c)). This interaction could account for altered function due to change in dynamic behavior for the local structural stability (Figure 3(c)). Electrostatic calculations were completed for further analysis (Figure 4). In particular, residues K624, E499, and Y532 are placed into different interactions (Figure 3(b)). Local ΔG per amino acid increases a net positive value corresponding to unfavorable stability in the pocket and longer dynamics would be required to determine the overall effect but can be expected to be destabilizing.

### 4.2. Dimer Formation Effects

Mapping electrostatics was accomplished using the Poisson–Boltzmann calculation for solvation on the entire 745 amino acid structure. The effects of the changes were strongly pronounced on electrostatic distribution with a +3 KT/E cutoff for both. The WT particle (all 745 aa) shows a distinct distribution of charge around K533, which shows few negatively charged regions and large neutral pockets due to the rich hydrophobic residues found in the helices mentioned above. The threonine mutation seems to change the distribution for the charges and places the position for the negative charges further away from each other while increasing the size of the neutrally charged regions (Figures 4(a) and 4(b)). The p.K533E local region is charged to match the positive lysine residue with adjacent glutamic acid residues (within 6 Å) (Figure 4(b)). Conversely, residue K533 has positive charges that are impacted by residues within 6 Å, namely E471, Q472, I474, I475, E499, Y532, P534, P535, and K624 (Figure 4(a)) [16–18, 20–23]. The variant p.K533E does not seem to bear any effect on the dimer itself (Figures 3 and 4), since the variant is distal to the interface between the monomers. However, there is significant disruption in the local vicinity where the lysine is natively inserted into a pocket with its nearby residues that are oppositely charged and engage in H-bonds and salt bridge interactions, which is lost with the negative charge from glutamic acid substitution (Figure 3).

## 5. Discussion

For patients with AMN, options for treatment are currently limited. However, recent research with mouse models poses a potential solution. In X-ALD patients, the buildup of VLCFAs is accompanied by increased levels of radical oxygen species. These radicals drive disease progression, damaging tissue and ultimately causing the appearance of neurological symptoms. Mice with X-ALD analogues have been successfully treated with a mixture of antioxidants, bringing radical levels under control and halting this pathogenic process [31]. If later trials continue to prove promising, it is possible that our proband and other AMN patients could one day be treated in a similar manner.

Although our proband presented with a poorly-understood variant in *ABCD1*, his case did not differ significantly from the typical AMN phenotype. Symptoms began in his late twenties, which fits neatly in the expected age range of 20 to 40 years old. AMN typically has spinal cord involvement, something that was confirmed by the proband's MRIs. His leg weakness and gait abnormality is characteristic of the disease, as is urinary incontinence. Vomiting and nausea are associated with adrenal insufficiency, while the proband's speech and mental abnormalities could be associated with cerebral changes [3]. Despite this, it must be said that a patient having the same *ABCD1* variant expressed by our proband could present with different symptoms. Pathogenic variants in *ABCD1* rarely result in predictable phenotypes—even when an identical version is possessed by family members [4].

The protein informatics derived from the statistical mechanics calculations applied to the molecular modeling for the variants versus the wild type gives us clear indication for dysfunction of the normal protein behavior at a molecular level that would impact the function. For instance, we found variant p.K533E causes a net increase in free energy of 2.321 kcal/aa∗mol∗Å^2^, which destabilizes the local region within 12 Å of residue K533. Due to the local structural change mitigated through backbone residues and secondary-structure interaction changes (H-bond network not shown) allow correlated motion to propagate throughout the structure further frustrating the proper formation of the dimer.

Due to the rarity of our proband's *ABCD1* variant, the commercial laboratory was not able to confidently name the variant pathogenic. Additionally, logistical challenges and the proband's small family size made family segregation analysis impractical. As a result, this process could not be completed to help us better understand the significance of his variant. Thankfully, molecular modeling provided us with another opportunity. Protein molecular modeling supported suspicions that this variant would impact protein function in a clinically significant way. It was without any suspicion that this variant would not have been classified pathogenic given the gross alteration to the structure, which was strongly supported by the PIP analysis.

## 6. Conclusion

In conclusion, we report a proband with a rare variant in *ABCD1*, c.1599G>T (p.Lys533Asn). The lack of information on this variant prevented the genetic testing company from classifying his variant as pathogenic, but the molecular charges seem to indicate this region of the protein is significantly perturbed from equilibrium (Figure 3(b)). Utilization of the protein modeling also provided information for incorporation into the proband's larger clinical picture. Additionally, one instance of this variant's pathogenicity is listed in the ALD Mutation Database. Combined, this evidence allowed for a confident diagnosis of AMN. Our proband had typical symptoms of AMN, but the phenotypic variability inherent in *ABCD1* could result in a different X-ALD presentation for other individuals possessing this variant.

## Data Availability

Datasets and materials are detailed in manuscript.

## Abbreviations

*ABCD1*: ATP-binding cassette, subfamily D, member 1 (gene)
VLCFA: Very long chain fatty acids
X-ALD: X-linked adrenoleukodystrophy
AMN: Adrenomyeloneuropathy.
